# Vericiguat combined with “new quadruple” therapy enhances cardiac function and life quality in patients with heart failure: a single-center prospective study

**DOI:** 10.3389/fcvm.2024.1476976

**Published:** 2024-10-11

**Authors:** Di Zhao, Yanjuan Zhang, Yonghong Yong, Liansheng Wang, Jiabao Liu

**Affiliations:** Department of Cardiology, The First Afﬁliated Hospital of Nanjing Medical University, Nanjing, China

**Keywords:** heart failure, Vericiguat, myocardial infarction, dilated cardiomyopathy, valvular heart disease

## Abstract

**Objective:**

To investigate the therapeutic effect of Vericiguat combined with “new quadruple” drugs on patients with heart failure (HF).

**Methods:**

From December 1, 2022 to February 1, 2024, 103 patients with heart failure were consecutively enrolled from the cardiology clinic or ward of the First Affiliated Hospital of Nanjing Medical University. Before enrollment, the patients’ left ventricular ejection fraction (LVEF), left ventricular end diastolic diameter (LVEDD), N-terminal pro-B-type natriuretic peptide (NT-proBNP), liver and kidney function electrolytes, and Minnesota Living with Heart Failure Questionnaire (MLHFQ) and other indicators were measured. Patients diagnosed with reduced ejection fraction (HFrEF) and with heart failure with mildly reduced ejection fraction (HFmrEF) were treated with Vericiguat combined with “ARNI, BB, MRA, SGLT2i” therapy. Patients diagnosed with preserved ejection fraction (HFpEF) were treated with Vericiguat combined with “ARNI, BB, SGLT2i” therapy. The above indicators were rechecked after 1 month of treatment.

**Results:**

For all patients, comparison after treatment: LVEF (38.1 ± 8.5% vs. 43.1 ± 8.5%, *P* < 0.01), LVEDD (60.5 ± 8.1 vs. 58.2 ± 7.3 mm, *P* < 0.01), NT-proBNP (4,567.8 ± 5,163.9 vs. 1,895.6 ± 2,702.1 ng/L, *P* < 0.01), MLHFQ (45.72 ± 11.09 vs. 32.29 ± 9.41, *P* < 0.01). Further subgroup analysis showed that Vericiguat combined with “ARNI, BB, SGLT2i or MRA” improved the LVEF and reduced NT-proBNP levels in patients with HFrEF, HFmrEF or HFpEF. and improved patients’ quality of life scores. The intergroup comparison showed the therapeutic effect of the combination was equivalent in HF caused by myocardial Infarction (MI), dilated cardiomyopathy (DCM) or Valvular Heart Disease (VHD).

**Conclusion:**

Vericiguat combined with the “new quadruple” therapy has a significant therapeutic effect on patients with heart failure caused by MI, DCM or VHD.

## 1 Introduction

Heart failure represents the advanced stage of various heart diseases, yet the field of cardiovascular medicine remains unconquered, earning it the title of “the cancerous frontier” within cardiology (1). Given its substantial disease burden, heart failure is poised to become a prominent battleground in future cardiovascular healthcare. The classification of heart failure is based on the differentiation of left ventricular ejection fraction (LVEF) and its response to treatment, resulting in distinct categories: heart failure with reduced ejection fraction (HFrEF), heart failure with improved ejection fraction (HFimpEF), heart failure with mildly reduced ejection fraction (HFmrEF), and heart failure with preserved ejection fraction (HFpEF). Specifically, HFrEF, LVEF ≤ 40%; HFmrEF, LVEF ranges from 40% to 49%; HFpEF, LVEF ≥ 50%. As a subgroup of HFrEF, with a previous LVEF ≤ 40% and a follow-up LVEF > 40% and an increase of ≥10% from baseline, it is defined as HFimpEF (2, 3).

The incidence of heart failure among adults in developed countries ranges from 1.0% to 2.0% (4, 5). According to a survey data in China from 2012 to 2015, the incidence of heart failure among adults aged ≥35 was 1.3%, which is approximately 13.7 million heart failure patients, an increase of 0.4% compared to 2000. The incidence rates of HFrEF, HFmrEF, and HFpEF are 0.7%, 0.3%, and 0.3%, respectively (6). The aging population in China is exacerbating, resulting in an escalating incidence of chronic conditions such as coronary atherosclerotic disease (CAD), hypertension, diabetes, and obesity. Additionally, advancements in medical care have extended the survival rates of patients with cardiac ailments. Collectively, these factors contribute to the prevailing prevalence of heart failure in China (7).

Myocardial infarction (MI), dilated cardiomyopathy (DCM), and valvular heart disease (VHD) currently represent prevalent etiologies of heart failure (2, 8). The emergence of drugs such as angiotensin receptor-neprilysin inhibitors (ARNI), beta blockers (BB), mineralocorticoid receptor antagonists (MRA), and sodium-glucose cotransporter-2 inhibitors (SGLT2i) has significantly enhanced cardiac function in patients with heart failure, earning them the designation of “new quadruple” therapy for heart failure treatment, which has been incorporated into the guidelines (7). However, there remains a subset of heart failure patients who fail to achieve sustained and effective improvement in cardiac function, necessitating frequent hospitalizations and treatments that may even result in mortality. Henceforth, it is imperative to explore novel therapeutic approaches and combination regimens for managing heart failure.

Vericiguat is a soluble guanylate cyclase (sGC) agonist that can directly stimulate guanylate cyclase in a nitric oxide-independent manner when there is insufficient nitric oxide, thereby increasing intracellular levels of cyclic guanosine monophosphate (cGMP). This leads to improvements in myocardial and vascular function, delays left ventricular remodeling, prevents or even reverses left ventricular hypertrophy, ultimately resulting in enhanced myocardial and vascular function (9–11). Its unique mechanism of action serves as the key breakthrough in heart failure treatment, which will further enhance the prognosis for patients with heart failure. Currently, there is a lack of comprehensive reports on the therapeutic efficacy of Vericiguat and new quadruple drugs (ARNI, BB, MRA, SGLT2i) in Chinese heart failure patients, necessitating further research. Therefore, the objective of this study is to assess the clinical effectiveness and safety of combining Vericiguat with “New Quadruple” drugs for treating heart failure resulting from MI, DCM and VHD, thereby providing valuable evidence for evidence-based medicine.

## 2 Methods

### 2.1 Design and eligibility

Inclusion criteria: (1) Meet the diagnostic criteria for heart failure in the Chinese Guidelines for the Diagnosis and Treatment of Heart Failure (2018 Edition); (2) Including heart failure caused by myocardial infarction, dilated cardiomyopathy, or valvular heart disease. (3) Despite receiving treatment with guideline-directed medical therapy (e.g., angiotensin receptor-neprilysin inhibitor, beta-blocker, sodium-glucose cotransporter-2 inhibitor, and mineralocorticoid receptor antagonist), there are patients who have recently experienced a deterioration in their heart failure. (4) Patients and their families were aware of and agreed to participate in the study. Exclusion criteria: (1) patients with primary valvular heart disease requiring surgery or intervention, or who was within 3 months after valvular surgery or intervention; (2) Rheumatic, alcoholic, and other secondary cardiomyopathy; (3) Autoimmune diseases; (4) Advanced tumors; (5) Severe sepsis; (6) Functional disorders of vital organs including the brain, liver, lungs, and kidneys; (7) Abnormal thyroid function; (8) Mental disorders impeding normal communication; (9) Individuals with malignant tumors and hematological disorders. This research protocol has been reviewed and approved by the Medical Ethics Committee of the First Affiliated Hospital of Nanjing Medical University (Approval No. 2022-SR-529). All patients provided informed consent and signed the corresponding consent forms. Patients with heart failure and reduced ejection fraction (HFrEF, LVEF ≤ 40%) and those with mildly reduced ejection fraction (HFmrEF, LVEF 41% to 49%) were treated with a new quadruple therapy of ARNI, BB, MRA, and SGLT2i, in addition to Vericiguat. Patients with heart failure and preserved ejection fraction (HFpEF), specifically those with LVEF ≥ 50% and increased left ventricular filling pressure, were treated with a combination of ARNI, BB, and SGLT2i, along with Vericiguat. Prior to enrollment, echocardiography was used to routinely assess the patient's cardiac function, including left ventricular systolic function (LVEF, LVEDD, LVESD) and diastolic function (E/A, E/e’), along with pertinent laboratory markers such as NT-proBNP, liver and kidney function electrolytes, and the Minnesota Heart Failure Quality of Life Scale (MLHFQ). After 1 month of treatment, the therapeutic effect was assessed, and the aforementioned parameters were re-evaluated.

### 2.2 Standard cardiac function evaluation

We utilized the Philip EPIQ-CV ultrasound instrument with a S5⁃1 probe (frequency range of 2.0–4.5 MHz). All patients were placed in a left lateral decubitus position. All images were ECG-triggered and stored in a cine-loop format with 3–5 cardiac cycles. Left atrial antero-posterior dimension (LAD), left ventricular dimension at diastole (LVDd) and left ventricular dimension at systole (LVDs) were assessed via M-mode in the parasternal long-axis view. Left ventricular ejection fraction (LVEF) was measured using modified biplane Simpson's method according to the ASE guidelines (12). All measurements were taken three times and the average value was calculated. Consistency verification: The intra- and interobserver variability for cardiac function was analyzed repeatedly in 15 randomly selected participants using Blant-Altman analysis. To assess intra-observer variability, one observer evaluated the same studies on two separate occasions. To assess interobserver variability, two independent observers performed the analyses separately.

### 2.3 Laboratory tests

Routine laboratory tests (complete blood count, routine urinalysis and serum chemistry profile) were performed in the local laboratories of the participating institutions. The estimated glomerular filtration rate (eGFR) was calculated according to the modified eGFR equation for Chinese patients. Plasma NT-proBNP levels were measured in the Department of Laboratory Medicine, the First Affiliated Hospital of Nanjing Medical University, Nanjing, China, using dedicated kit-based NT-proBNP assays (Roche Diagnostics, Basel, Switzerland).

### 2.4 Evaluation of quality of life in patients with heart failure

Minnesota Living with Heart Failure Questionnaire (MLHFQ) was used to assess patients’ quality of life before and after treatment, with higher scores indicating poorer quality of life. Adverse reactions during treatment were to be observed and recorded in both patient groups. The MLHFQ was a standardized instrument utilized for evaluating the quality of life in individuals with heart failure (13), comprising 21 questions that offer diverse response options to gauge the degree to which heart failure impacts an individual's daily activities.

### 2.5 Statistical analysis

Statistical analysis was performed using SPSS 22.0 software. Count data were presented as numbers of cases, with inter-group comparisons using chi-square tests. Quantitative data were expressed as mean ± standard deviation $\left(\bar{x}\pm s\right)$, and when the data were normally distributed and showed homogeneity of variance, paired sample *t*-tests were used to compare before and after treatment within the same patient. Multiple group comparisons were conducted using one-way ANOVA and the LSD *t*-test for multiple comparisons. And the Bonferroni correction was employed to control for Type I errors. Shapiro-Wilk test was used to check for normality. Missing data was managed using multiple imputation and exclusion method. The overall, intra-group, and inter-group diversity levels were calculated with a Bonferroni correction for all significance levels. *P* < 0.05 denoted a statistically significant difference. All *P*-values were two-tailed.

## 3 Results

### 3.1 General information and medications for heart failure

From December 1, 2022 to February 1, 2024, a consecutive series of 103 heart failure patients, comprising 81 males and 22 females, were recruited at the Cardiology Department's outpatient and inpatient facilities of the First Affiliated Hospital of Nanjing Medical University. This study included a total of 103 patients with heart failure, specifically comprising 31 with post-myocardial infarction heart failure, 38 with dilated cardiomyopathy-related heart failure, and 34 with valvular heart failure. The youngest patient was 30 years old, the oldest 77 years, with an average age of 56.2 years. The patients’ general information and medication details were presented in Table 1. Continuous variables are expressed as mean ± SD, and categorical variables as [*n* (%)]. The mean age of patients with heart failure due to DCM was significantly lower compared to those with heart failure following MI and VHD, indicating that individuals with DCM experienced the onset of heart failure at an earlier stage in life, aligning with previous literature findings (14). The majority of patients consisted of individuals with HFmrEF and HFrEF, with HFrEF patients representing the largest proportion. The majority of patients enrolled in this study were classified as NYHA functional class II-III. The drug Vericiguat was administered to all enrolled patients.

**Table 1:** Patients’ basic information and medication use.

Characteristics	Heart failure following MI	heart failure following DCM	heart failure following VHD	All
Cases (*n*)	31	38	34	103
Gender [male (%)]	26 (83.87)	31 (81.58)	24 (70.59)	81 (78.64)
Age (years)	62 ± 9	48 ± 13	60 ± 13	56 ± 13
Height (cm)	166.89 ± 7.61	167.13 ± 6.26	167.20 ± 7.39	167.08 ± 7.11
Weight (kg)	65.88 ± 14.02	70.12 ± 13.19	69.62 ± 12.45	68.67 ± 13.21
Systolic BP (mmHg)	117.55 ± 17.23	117.76 ± 14.26	115.03 ± 16.10	116.79 ± 5.70
Diastolic BP (mmHg)	74.42 ± 9.66	76.89 ± 8.94	73.85 ± 9.00	75.15 ± 9.19
Heart rate (beats/min)	81.87 ± 11.60	76.61 ± 14.17	78.65 ± 12.71	78.86 ± 13.01
Medical history				
Hypertension [*n* (%)]	11 (35.48%)	11 (28.95%)	12 (35.29%)	34 (33.01%)
Diabetes mellitus [*n* (%)]	6 (19.35%)	5 (13.16%)	5 (14.7%)	16 (15.53%)
NYHA functional class				
I	0 (0%)	0 (0%)	0 (0%)	0 (0%)
II	16 (51.61%)	16 (42.11%)	14 (41.18%)	46 (44.66%)
III	13 (41.94%)	20 (52.63%)	19 (55.88%)	52 (50.49%)
IV	2 (6.45%)	2 (5.26%)	1 (2.94%)	5 (4.85%)
Heart failure classification by ejection fraction				
HfpEF	5 (16.13%)	5 (13.16%)	1 (2.94%)	11 (10.68%)
HFmrEF	12 (38.71%)	11 (28.95%)	6 (17.65%)	29 (28.16%)
HFrEF	14 (45.16%)	22 (57.89%)	27 (79.41%)	63 (61.17%)
Medication				
Angiotensin receptor-neprilysin inhibitors	27 (87.10%)	35 (92.11%)	30 (88.24%)	92 (89.32%)
Beta-blockers	26 (83.87%)	34 (89.47%)	29 (85.29%)	89 (86.40%)
Furosemide	26 (83.87%)	31 (81.58%)	28 (82.35%)	85 (82.52%)
Mineralocorticoid receptor antagonists	27 (87.10%)	33 (86.84%)	30 (88.23%)	90 (87.38%)
SGLT2i	28 (90.32%)	34 (89.47%)	31 (91.18%)	93 (90.29%)
Vericiguat	31 (100%)	38 (100%)	34 (100%)	103 (100%)

### 3.2 The impact of Vericiguat in combination with “new quadruple” drugs on cardiac function, electrolyte levels, and MLHFQ in patients with heart failure

The data were normally distributed and showed homogeneity of variance. The data of patients were compared before and after treatment, utilizing a paired samples *T*-test for analysis. The assessment of cardiac function, NT-proBNP levels, hepatic and renal function, electrolyte balance, and MLHFQ scores in heart failure patients treated with Vericiguat combined with the “new quadruple” regimen for 1 month demonstrated significant improvements in LAD, LVESD, LVEDD, LVEF, NT-proBNP levels, and MLHFQ scores compared to pre-treatment values (*P* < 0.01). However, there were no significant differences observed in ALT, AST, creatinine levels, urea nitrogen levels or liver and kidney functions before and after treatment (*P* > 0.05). These findings are summarized in Table 2.

**Table 2:** Effects of Vericiguat combined with “new quadruple” on cardiac function, electrolytes and MLHFQ in overall heart failure patients (*n* = 103).

Variables	Prior to medication	After medication	*t*	*P*
LAD (mm)	43.37 ± 7.13	41.84 ± 6.66	5.244	<0.01
LVESD (mm)	49.01 ± 8.762	45.75 ± 8.407	7.791	<0.01
LVEDD (mm)	60.51 ± 8.07	58.22 ± 7.30	5.876	<0.01
LVEF (%)	38.13 ± 8.47	43.08 ± 8.55	−8.243	<0.01
NT-proBNP (ng/L)	4,567.80 ± 5,163.93	1,895.63 ± 2,702.12	5.735	<0.01
ALT	26.80 ± 16.92	29.94 ± 18.34	−1.071	0.291
AST	25.80 ± 11.71	28.17 ± 9.16	−1.206	0.235
Creatinine	93.52 ± 38.03	98.19 ± 51.18	−1.220	0.230
Urea nitrogen	7.76 ± 4.57	7.72 ± 3.55	0.079	0.938
K^+^	3.96 ± 0.45	4.10 ± 0.38	−1.770	0.084
Na^+^	140.25 ± 3.02	139.17 ± 2.77	1.851	0.072
Cl^−^	103.99 ± 3.82	103.17 ± 3.21	1.058	0.296
Ca^2+^	2.27 ± 0.15	2.30 ± 0.16	−1.118	0.270
MLHFQ	45.72 ± 11.09	32.29 ± 9.41	21.433	<0.01

### 3.3 Effects of Vericiguat combined with the “new quadruple” on cardiac function, electrolyte levels, and the Minnesota Living with Heart Failure Questionnaire (MLHFQ) in patients experiencing heart failure following myocardial infarction

The data were normally distributed and showed homogeneity of variance, the paired sample *t*-test was employed to compare pre- and post-treatment data in the subgroup analysis. Subgroup analysis revealed that, following 1 month of treatment in patients with post-myocardial infarction heart failure using Vericiguat in combination with the “new quadruple” drug, there was a significant improvement in left ventricular cavity size and cardiac function-related parameters, including LAD, LVESD, LVEDD, LVEF, NT-proBNP levels and MLHFQ scores when compared to pre-treatment values (*P* < 0.01), as presented in Table 3.

**Table 3:** Effects of Vericiguat combined with the “new quadruple” on cardiac function, NT-proBNP, and MLHFQ in patients with heart failure after myocardial infarction (*n* = 31).

Variables	Prior to medication	After medication	*t*	*P*
LAD (mm)	41.19 ± 4.39	40.19 ± 5.13	2.856	<0.01
LVESD (mm)	45.97 ± 7.40	43.97 ± 7.40	3.954	<0.01
LVEDD (mm)	57.94 ± 6.57	56.42 ± 6.41	3.239	<0.01
LVEF (%)	40.81 ± 8.36	43.87 ± 8.19	−3.648	<0.01
NT-proBNP (ng/L)	5,169.83 ± 5,427.62	1,964.16 ± 2,811.93	2.945	<0.01
MLHFQ	47.03 ± 11.16	34.94 ± 8.03	10.866	<0.01

**Figure 1:**
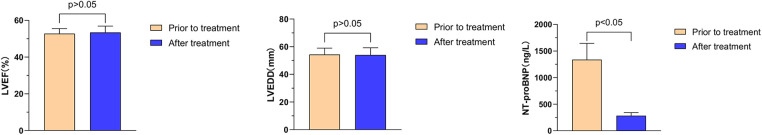
Illustrates the impact of Vericiguat in combination with ARNI, BB, and SGLT2i on cardiac function among patients with hFpEF following myocardial infarction. Effects of Vericiguat combined with ARNI, BB and SGLT2i on cardiac function in patients with HFpEF after MI (*n* = 5). LVEF before and after 1 month of treatment was 52.80% ± 2.72% vs. 53.43% ± 3.45%, *P* > 0.05; LVEDD (54.33 ± 4.62 vs. 54.02 ± 5.20 mm, *P* > 0.05); NT-proBNP (1,337.0 ± 306.9 vs. 283.6 ± 57.8 ng/L, *P* < 0.05).

Further analysis showed that the effects of Vericiguat combined with ARNI, BB and SGLT2i on cardiac function in patients with HFpEF after myocardial infarction were compared with LVEF (52.8% ± 0.7% vs. 53.4% ± 0.5%, *P* > 0.05) 1 month before and after treatment. LVEDD (54.3 ± 4.6 vs. 54.0 ± 5.2 mm, *P* > 0.05); NT-proBNP (1,337.0 ± 306.9 vs. 283.6 ± 57.8 ng/L, *P* < 0.05), as shown in Figure 1.

**Figure 2:**
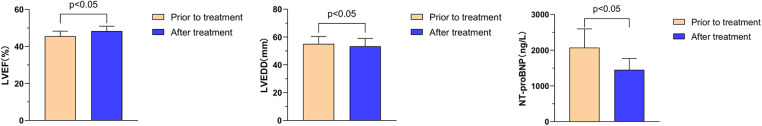
Effects of Vericiguat combined with ARNI, BB, MRA and SGLT2i on cardiac function in patients with hFmrEF after MI. The combination of Vericiguat with ARNI, BB, MRA, and SGLT2i demonstrated improved cardiac function in patients with HFmrEF after MI (*n* = 12). Following 1 month of treatment, there were significant improvements observed in LVEF (45.56% ± 2.69% vs. 48.31% ± 2.67%, *P* < 0.05), LVEDD (55.07 ± 5.31 vs. 53.36 ± 5.61 mm, *P* < 0.05), and NT-proBNP levels (2,073.9 ± 524 vs. 1,450.9 ± 314.5 ng/L, *P* < 0.05).

The effects of Vericiguat combined with ARNI, BB, MRA, and SGLT2i on cardiac function in patients with HFmrEF after myocardial infarction were compared before and after 1 month of treatment. The results showed a significant improvement in LVEF from 45.6% ± 2.7% to 48.3% ± 2.7% (*P* < 0.05), a reduction in LVEDD from 55.1 ± 5.3 to 53.4 ± 5.6 mm (*P* < 0.05), and a decrease in NT-proBNP levels from 2,073.9 ± 524.3 to 1,450.9 ± 314.5 ng/L (*P* < 0.05), as illustrated in Figure 2.

**Figure 3:**
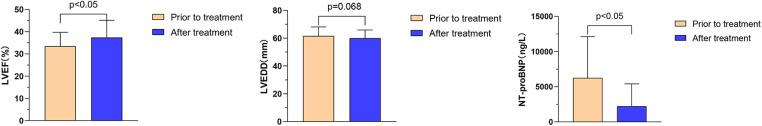
Effect of Vericiguat combined with ARNI, BB, MRA and SGLT2i on cardiac function in patients with hFrEF after MI. The combination of ARNI, BB, MRA, and SGLT2i improved cardiac function in HFrEF patients after MI (*n* = 14). Comparison before and after 1 month of treatment: LVEF (33.49% ± 6.23% vs. 37.39% ± 7.75%, *P* < 0.05); LVEDD (61.57 ± 6.48 vs. 60.0 ± 5.83 mm, *P* = 0.068); NT-proBNP (6,263.2 ± 5,846.4 vs. 2,232.5 ± 3,182.9 ng/L, *P* < 0.05).

The effects of Vericiguat combined with ARNI, BB, MRA, and SGLT2i on cardiac function in patients with HFrEF after myocardial infarction were compared before and after 1 month of treatment. The results showed that there was a significant improvement in LVEF (33.5% ± 6.2% vs. 37.4% ± 7.8%, *P* < 0.05), a slight decrease in left ventricular end-diastolic diameter (LVEDD) (61.6 ± 6.5 vs. 60.0 ± 5.8 mm, *P* = 0.068), and a significant reduction in NT-proBNP levels (6,263.2 ± 5846.4 vs. 2,232.5 ± 3,182.9 ng/L, *P* < 0.05), as illustrated in Figure 3.

### 3.4 Effects of Vericiguat combined with the “new quadruple” on cardiac function, NT-proBNP and MLHFQ in patients with heart failure in dilated cardiomyopathy

The data were normally distributed and showed homogeneity of variance, the paired sample *t*-test was employed to compare pre- and post-treatment data in the subgroup analysis. Subgroup analysis revealed that following 1 month of treatment with Vericiguat combined with the “new quadruple” drug, patients with dilated cardiomyopathy and heart failure demonstrated significant improvements in left ventricular cavity size and scores of cardiac function indicators (including LAD, LVESD, LVEDD, LVEF), as well as NT-proBNP levels and MLHFQ scores when compared to pre-treatment values (<0.01). These findings are summarized in Table 4. The results were not overgeneralized across different subgroups as further examination through subgroup analysis did not reveal any interaction between the factors.

**Table 4:** Effects of Vericiguat combined with “new quadruple” on cardiac function and MLHFQ in patients with DCM heart failure (*n* = 38).

Variables	Prior to treatment	After treatment	*t*	*P*
LAD (mm)	41.21 ± 7.29	39.34 ± 5.44	3.501	<0.01
LVESD (mm)	49.66 ± 9.66	45.66 ± 9.21	4.816	<0.01
LVEDD (mm)	61.55 ± 8.09	58.87 ± 7.38	4.055	<0.01
LVEF (%)	38.42 ± 9.25	44.50 ± 9.28	−6.292	<0.01
NT-proBNP (ng/L)	2,232.35 ± 2,484.26	856.88 ± 1,452.81	3.575	<0.01
MLHFQ	48.32 ± 9.94	34.58 ± 9.31	14.561	<0.01

**Figure 4:**
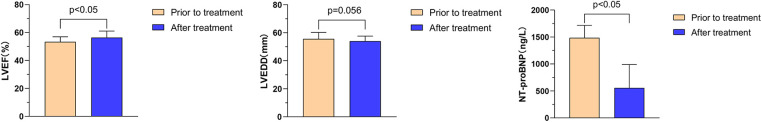
Effect of Vericiguat combined with ARNI, BB and SGLT2i on cardiac function in DCM hFpEF patients. The combination of Vericiguat with ARNI, BB, and SGLT2i improved cardiac function in DCM HFpEF patients (*n* = 5). Comparison before and after 1-month treatment: LVEF (53.36% ± 3.58% vs. 56.44% ± 4.69%, *P* < 0.05); LVEDD (55.60 ± 4.62 vs. 54.00 ± 3.54 mm, *P* = 0.056); NT-proBNP (1,484.3 ± 231 vs. 556.1 ± 432.3 ng/L, *P* < 0.01).

Further analysis showed that the effects of Vericiguat combined with “ARNI, BB, SGLT2i” on cardiac function in patients with DCM HFpEF were compared before and after 1 month of treatment: LVEF (53.4% ± 3.6% vs. 56.4% ± 4.7%, *P* < 0.05); LVEDD (55.6 ± 4.6 vs. 54.0 ± 3.5 mm, *P* = 0.056); NT-proBNP (1,484.3 ± 231 vs. 556.1 ± 432.3 ng/L, *P* < 0.01), as shown in Figure 4.

**Figure 5:**
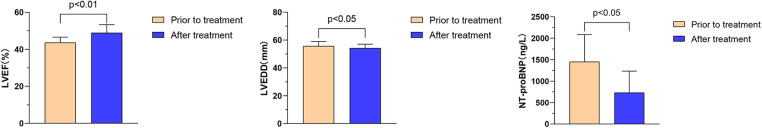
Effects of Vericiguat combined with ARNI, BB, MRA, SGLT2i on cardiac function in DCM hFmrEF patients. Vericiguat combined with ARNI, BB, MRA, and SGLT2i improved cardiac function in DCM HFmrEF patients (*n* = 11). LVEF (43.67% ± 2.89% vs. 48.97% ± 4.36%, *P* < 0.01) was compared before and after 1 month of treatment. LVEDD (55.73 ± 3.23 vs. 54.27 ± 2.73 mm, *P* < 0.05); NT-proBNP (1,455.6 ± 630.9 vs. 735.3 ± 498.7 ng/L, *P* < 0.05).

The effect of combination therapy with ARNI, BB, MRA, SGLT2i on cardiac function in DCM HFmrEF patients (*n* = 11). Comparison before and after treatment for 1 month: LVEF (43.7% ± 2.9% vs. 48.9% ± 4.4%, *P* < 0.01); LVEDD (55.7 ± 3.2 vs. 54.3 ± 2.7 mm, *P* < 0.05); NT proBNP (1,455.6 ± 630.9 vs. 735.3 ± 498.7 ng/L, *P* < 0.05), as shown in Figure 5.

**Figure 6:**
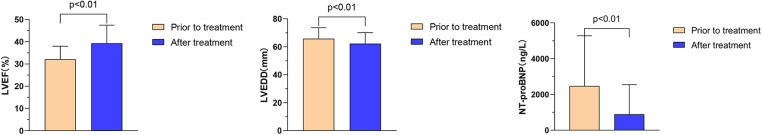
Effect of Vericiguat combined with ARNI, BB, MRA, SGLT2i on cardiac function in patients with DCM hFrEF. Vericiguat combined with ARNI, BB, MRA, SGLT2i improved cardiac function in patients with DCM HFrEF (*n* = 22). Before and after 1 month of treatment, LVEF (32.12% ± 5.87% vs. 39.31% ± 8.13%, *P* < 0.01); LVEDD (65.82 ± 7.82 vs. 62.27 ± 7.82 mm, *P* < 0.01); NT-proBNP (2,475.1 ± 2,803.3 vs. 894.9 ± 1,655.7 ng/L, *P* < 0.01).

The effects of Vericiguat combined with ARNI, BB, MRA, SGLT2i on the cardiac function of DCM HFrEF patients (*n* = 22) were compared before and after 1 month of treatment: LVEF (32.1% ± 5.9% vs. 39.3% ± 8.1%, *P* < 0.01); LVEDD (65.8 ± 7.8 vs. 62.3 ± 7.8 mm, *P* < 0.01); NT-proBNP (2,475.1 ± 2,803.3 vs. 894.9 ± 1,655.7 ng/L, *P* < 0.01), as shown in Figure 6.

### 3.5 Effects of Vericiguat combined with “new quadruple” on cardiac function, NT-proBNP and MLHFQ in patients with VHD heart failure

The data were normally distributed and showed homogeneity of variance, the paired sample *t*-test was employed. Subgroup analysis revealed significant improvements in LAD, LVESD, LVEDD, LVEF, NT-proBNP levels, and MLHFQ scores among patients with VHD heart failure after 1 month of treatment with Vericiguat combined with the “new quadruple” drugs compared to pre-treatment values (*p* < 0.01). This was illustrated in Table 5.

**Table 5:** Effects of Vericiguat combined with “new quadruple” on cardiac function, NT-proBNP and MLHFQ in patients with VHD heart failure (*n* = 34).

Variables	Prior to treatment	After treatment	*t*	*P*
LAD (mm)	47.76 ± 7.11	46.15 ± 7.14	2.856	<0.01
LVESD (mm)	51.06 ± 8.33	47.47 ± 8.21	5.078	<0.01
LVEDD (mm)	61.71 ± 8.92	59.15 ± 7.86	3.129	<0.01
LVEF (%)	35.27 ± 6.87	40.74 ± 7.74	−4.467	<0.01
NT-proBNP (ng/L)	6,027.51 ± 6,002.50	2,662.93 ± 3,165.57	4.086	<0.01
MLHFQ	41.62 ± 11.39	27.32 ± 8.95	11.841	<0.01

**Figure 7:**
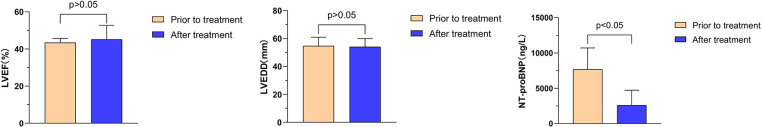
Effects of Vericiguat combined with ARNI, BB and SGLT2i on cardiac function in patients with VHD HFmrEF. Effects of Vericiguat combined with ARNI, BB and SGLT2i on cardiac function in patients with VHD HFmrEF (*n* = 6). Comparison of LVEF before and after 1 month of treatment showed no significant difference (43.47% ± 2.25% vs. 45.21% ± 7.52%, *P* > 0.05). Similarly, there was no significant difference observed in LVEDD (54.71 ± 6.16 vs. 54.14 ± 5.82 mm, *P* > 0.05). However, a significant decrease was noted in NT-proBNP levels following treatment (7,704.6 ± 3,015 vs. 2,613.4 ± 2,108.6 ng/L, *P* < 0.05).

Further analysis reveals that the combination of Vericiguat with ARNI, BB, MRA, and SGLT2i significantly impacts heart function in patients with HFmrEF of VHD (*n* = 6). Comparison before and after 1 month of treatment: LVEF (43.5% ± 2.3% vs. 45.2% ± 7.5%, *P* > 0.05); LVEDD (54.7 ± 6.2 vs. 54.1 ± 5.8 mm, *P* > 0.05); NT-proBNP (7,704.6 ± 3,015.5 vs. 2,613.4 ± 2,108.6 ng/L, *P* < 0.05), as depicted in Figure 7.

**Figure 8:**
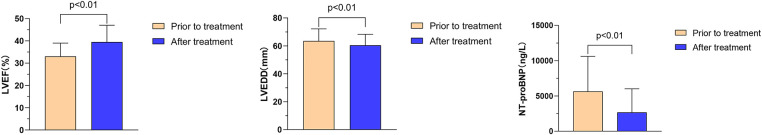
Effect of Vericiguat combined with ARNI, BB, MRA, SGLT2i on cardiac function in patients with VHD hFrEF. The effect of Vericiguat combined with ARNI, BB, MRA, and SGLT2i on cardiac function in patients with VHD HFrEF was improved (*n* = 27). LVEF (33.07% ± 5.94% vs. 39.53% ± 7.48%, *P* < 0.01) was compared before and after 1 month of treatment. LVEDD (63.52 ± 8.69 vs. 60.44 ± 7.89 mm, *P* < 0.01); NT-proBNP (5,646.3 ± 4,961.3 vs. 2,674.2 ± 3,330.2 ng/L, *P* < 0.01).

The effects of Vericiguat combined with ARNI, BB, MRA, and SGLT2i on cardiac function were compared in patients with VHD HFrEF before and after 1 month of treatment. The results showed significant improvements in the following parameters: left ventricular ejection fraction (LVEF) increased from 33.1% ± 5.9% to 39.5% ± 7.5% (*P* < 0.01), left ventricular end-diastolic diameter (LVEDD) decreased from 63.5 ± 8.7 to 60.4 ± 7.9 mm (*P* < 0.01), and NT-proBNP levels decreased from 5,646.3 ± 4,961.3 to 2,674.2 ± 3,330.2 ng/L (*P* < 0.01), as illustrated in Figure 8.

### 3.6 The efficacy of Vericiguat combined with the “new quadruple” in patients with heart failure due to myocardial infarction, dilated cardiomyopathy, and valvular heart disease was compared

The data were normally distributed and show homogeneity of variance. The independent samples *t*-test was employed to compare the two groups. Multiple group comparisons were conducted using one-way ANOVA and the LSD *t*-test for multiple comparisons. And the Bonferroni correction was employed to control for Type I errors. Comparative analysis of therapeutic outcomes for heart failure with preserved ejection fraction (HFpEF): In this study, five patients with post-MI heart failure and five with DCM heart failure were included. No significant statistical difference was observed in the LVEF and LVEDD levels between the two groups prior to treatment (*P* > 0.05). Similarly, no significant difference was found in the LVEF and LVEDD levels between the two groups after 1 month of treatment (*P* > 0.05), suggesting that the combination of Vericiguat and the “New Quadruple” offers equivalent therapeutic efficacy in treating HFpEF resulting from MI and DCM.

The treatment effect was compared among patients with HFmrEF, including 12 patients with post-MI heart failure, 11 patients with DCM-related heart failure, and 6 patients with VHD failure. There were no statistically significant differences in LVEF and LVEDD levels among the three groups before treatment (*P* > 0.05). Similarly, after 1 month of treatment, there were no significant differences in LVEF and LVEDD levels among the three groups (*P* > 0.05), indicating that the combination of Vericiguat and the “New Quadruple” had an equivalent therapeutic effect on HFmrEF patients with post-MI, DCM-related and VHD-related heart failure.

Comparative Analysis of Therapeutic Outcomes for HFrEF: In this study, 14 patients with post-MI, 21 with DCM, and 26 with VHD heart failure were included. No significant statistical difference was observed in the LVEF and LVEDD levels among the three groups prior to treatment (*P* > 0.05). Similarly, no significant difference was found in the LVEF and LVEDD levels among the three groups after 1 month of treatment (*P* > 0.05), suggesting that the combination of Vericiguat and the “New Quadruple” offers probably equivalent therapeutic efficacy in treating HFrEF resulting from post-MI, DCM, and VHD. As shown in Table 6.

**Table 6:** Comparison of therapeutic effects of Vericiguat combined with “new quadruple” on patients with heart failure following MI, DCM, and VHD.

Classification based on LVEF	Group	LVEF (%)		LVEDD (mm)	
		Prior to treatment $\left(\bar{x}\pm s\right)$	After treatment $\left(\bar{x}\pm s\right)$	Prior to treatment $\left(\bar{x}\pm s\right)$	After treatment $\left(\bar{x}\pm s\right)$
HFpEF	HF following MI (*n* = 5)	52.80 ± 2.72	53.43 ± 3.45	54.33 ± 4.619	54.02 ± 5.20
	HF following DCM (*n* = 5)	53.36 ± 3.58	56.44 ± 4.69	55.60 ± 4.62	54.00 ± 3.54
	*t*	0.279	1.156	0.435	0.007
	*P*	0.789	0.281	0.675	0.995
HFmrEF	HF following MI (*n* = 12)	45.56 ± 2.69	48.31 ± 2.67	55.07 ± 5.31	53.36 ± 5.61
	HF following DCM (*n* = 11)	43.67 ± 2.89	48.97 ± 4.36	55.73 ± 3.23	54.27 ± 2.73
	HF following VHD (*n* = 6)	43.47 ± 2.25	45.21 ± 7.52	54.71 ± 6.16	54.14 ± 5.82
	*F*	1.876	1.368	0.101	0.117
	*P*	0.173	0.272	0.905	0.890
HFrEF	HF following MI (*n* = 14)	33.49 ± 6.23	37.39 ± 7.75	61.57 ± 6.48	60.00 ± 5.83
	HF following DCM (*n* = 22)	32.12 ± 5.87	39.31 ± 8.13	65.82 ± 7.82	62.27 ± 7.82
	HF following VHD (*n* = 26)	33.07 ± 5.94	39.53 ± 7.48	63.52 ± 8.69	60.44 ± 7.89
	*F*	0.261	0.377	1.276	0.518
	*P*	0.771	0.687	0.287	0.598

## 4 Discussion

In this real-world study, it was firstly observed that the combination of Vericiguat with “new quadruple” drugs exhibited significant therapeutic efficacy in patients suffering from heart failure following myocardial infarction, dilated cardiomyopathy, and valvular heart disease in Chinese people. This treatment approach demonstrated improvements in left ventricular ejection fraction (LVEF), reduction in left ventricular end-diastolic diameter (LVEDD), elevation of MLHFQ score, decrease in plasma NT-proBNP levels, and sustained effects even after 1 month of drug administration. Notably, no adverse effects on liver function, renal function or electrolyte balance were observed.

Heart failure is a complex clinical syndrome resulting from abnormal changes in cardiac structure and/or function caused by various factors, leading to ventricular contraction and/or diastolic dysfunction. Its main manifestations include dyspnea, fatigue, and fluid retention (such as pulmonary congestion, systemic congestion, and peripheral edema) (15). After coronary heart disease, heart failure has become the second most common cause of death related to heart disease (16). Although “new quadruple” drugs for heart failure such as ACEI/ARB/ARNI (Benazepril, Valsartan, Sacubitril/valsartan, etc.), beta blockers (Metoprolol, Bisoprolol), aldosterone receptor antagonists (Spironolactone), SGLT2 inhibitors (Engliflozin, Dagliflozin, etc.) have brought hope to the treatment of patients with heart failure (7), many patients still experience poor control every year requiring repeated hospitalization or even leading to death.

The VICTORIA study indicated that Vericiguat had good safety and tolerability, offering promising prospects for the long-term treatment of HFrEF patients experiencing worsening conditions (9). However, the therapeutic efficacy of Vericiguat in combination with the “new quadruple” regimen has not been extensively reported in Chinese heart failure patients. In this study, we observed a significant increase in the mean left ventricular ejection fraction from 38.1% to 43.1%, accompanied by a reduction in the left ventricular end-diastolic diameter from 60.5 to 58.2 mm, among patients with heart failure caused by myocardial infarction (MI), dilated cardiomyopathy (DCM), and valvular heart disease (VHD) after 1 month of treatment with Vericiguat combined with “new quadruple” drugs. Additionally, there was a notable decrease in the mean NT-proBNP levels from 4,567.8 to 1,895.6 ng/L, along with an improvement in the Minnesota Heart Dysfunction Life Quality Scale index (MLHFQ) scores from 45.72 to 32.29. The NO-sGC-cGMP pathway has been identified as a pivotal regulatory factor in the cardiovascular system, according to research reports. In pathological conditions, impairment of this pathway results in detrimental effects on target organs such as the heart (11). Vericiguat is an oral soluble guanylate cyclase (sGC) stimulant that directly activates sGC independent of nitric oxide (NO) concentration. It also enhances the sensitivity of NO, stimulates sGC to produce cGMP through a dual mechanism, exerts anti-inflammatory and anti-myocardial fibrosis effects, and subsequently improves vascular endothelial function. Additionally, it enhances the functionality of target organs such as the heart and kidneys (17). The findings of this study demonstrated that the combination of Vericiguat with “ARNI, BB, MRA, SGLT2i” resulted in an increased left ventricular ejection fraction in patients with HFrEF, HFmrEF, and HFpEF caused by MI, DCM, and VHD. Additionally, it led to a reduction in NT-proBNP levels and improvement in patients’ quality of life scores. These results suggest that Vericiguat exerts therapeutic effects on the hearts of HFrEF patients through the NO-sGC-cGMP pathway while also exhibiting reparative properties for the hearts of HFpEF and HFmrEF patients.

In the past, there was a lack of understanding regarding the pathologic mechanisms underlying heart failure with preserved ejection fraction (HFpEF) and a dearth of treatment options for patients suffering from this type of cardiac condition. The SOCRATES-PRESERVED Study (18) conducted a randomized controlled trial involving 477 HFpEF patients, utilizing changes in NT-proBNP levels and echocardiographic left atrial volume (LAV) compared to baseline measurements after 12 weeks as the primary endpoint. An exploratory assessment of patients’ quality of life using disease-specific scales such as the Kansas City Cardiomyopathy Questionnaire (KCCQ) and the European Five-Dimensional Health Scale (EQ-5D) demonstrated that Vericiguat was well-tolerated and did not significantly alter NT-proBNP or LAV at 12 weeks when compared to placebo; however, it was associated with improved quality of life among HFpEF patients. Despite these negative findings contradicting initial expectations, given the encouraging results related to enhancing quality of life, the VITALITY-HFpEF Study was conducted in 2020 (19), encompassing a larger sample size, an extended observation period, and higher target dosages. Nevertheless, treatment with Vericiguat following 24 weeks of standard heart failure therapy failed to improve quality of life based on KCCQ scores when compared to placebo. This outcome may be attributed to adverse effects such as transient hypotension and decreased hemoglobin levels resulting from administration of higher doses (15 mg qd) of Vericiguat. These side effects caused additional physical discomfort among patients experiencing milder symptoms of heart failure, thereby potentially inflating their KCCQ scores. Alternatively, this could indicate that insufficient cyclic guanosine monophosphate (cGMP) is not a pivotal mechanism driving HFpEF progression relative to heart failure with reduced ejection fraction (HFrEF), necessitating further investigation in future studies. Due to the prolonged treatment required for patients with heart failure, the addition of any medication poses a risk of disrupting the delicate balance. Therefore, there is significant interest in exploring the potential of Vericiguat as a novel treatment for heart failure when combined with other drugs. The present study innovatively employed Vericiguat in combination with “new quadruple” drugs to investigate the efficacy and safety of this treatment approach in patients with HFpEF. Despite the limited number of included HFpEF patients, the findings from this study provided confirmation that the use of Vericiguat combined with the “new quadruple” regimen was both safe and effective for HFpEF patients. The future will witness an increased enrollment of HFpEF patients in studies, aiming to further assess the efficacy of Vericiguat combined with the “new quadruple” for treating this specific type of heart failure.

Rapid initiation of GDMT (Guideline directed medical therapy) is crucial for pharmacological management of heart failure (20). Additionally, our study revealed that for heart failure patients with hypotension, GDMT anti-heart failure drugs that do not impact blood pressure, such as Vericiguat, could be initiated initially. Subsequently, other GDMT anti-heart failure medications could be gradually introduced as the patient's cardiac function improves and blood pressure stabilizes. The administration of Vericiguat can be initiated in patients with acute heart failure following a cessation of intravenous medications for a minimum duration of 24 h (21). Based on our treatment experience, a combination approach utilizing maximum extent of GDMT drugs yielded more pronounced improvement in heart failure patients. This suggested that the pathogenesis of heart failure involves multiple pathophysiological systems and distinct molecular pathways. Only through combining GDMT drugs with diverse mechanisms of action can we effectively target the pathological molecular pathways from various angles and targets to achieve optimal therapeutic outcomes (22). Furthermore, our findings indicated that simultaneous and consistent administration of Vericiguat and “new quadruple” drugs resulted in maximum therapeutic efficacy when all five drugs were used together. It was worth noting that poor treatment compliance leading to drug discontinuation or dosage reduction significantly hampered treatment effectiveness. Therefore, enhancing patient adherence to medication regimens and ensuring continuous drug intake were paramount in managing heart failure.

### 4.1 Limitations

Owing to the small sample size, particularly the limited number of patients in the HFpEF group, there may be a potential bias in the statistical results. Therefore, further validation is required using data from large-scale, multi-center clinical studies.

In conclusion, this study suggested that the combination of Vericiguat and the “new quadruple” regimen could significantly enhance cardiac function and improve quality of life in patients with heart failure caused by myocardial infarction (MI), dilated cardiomyopathy (DCM), and valvular heart disease (VHD). Additionally, it effectively inhibited ventricular remodeling while maintaining comparable safety and efficacy. This study may provide valuable data support and serves as a reference for the treatment of patients with clinical heart failure.

## Data Availability

The original contributions presented in the study are included in the article/Supplementary Material, further inquiries can be directed to the corresponding authors.
